# Three-dimensional analysis of miniscrew position changes during bone-borne expansion in young and late adolescent patients

**DOI:** 10.1186/s40510-023-00469-0

**Published:** 2023-06-05

**Authors:** Marco Migliorati, Anna De Mari, Fabio Annarumma, Hussein Aghazada, Giovanni Battista, Alessandra Campobasso, Maria Menini, Antonino Lo Giudice, Lucia H. S. Cevidanes, Sara Drago

**Affiliations:** 1 Orthodontic Department, Genova University, Largo R. Benzi 10, 16100 Genoa, Italy; 2 Private Practice, Piazza Luigi Rizzo 1, Ciampino, RM Italy; 3 Private Practice, Piazzale Ardeatino, 1G, 00154 Rome, RM Italy; 4 Private Practice, Foggia, FG Italy; 5 University of Foggia, Via Rovelli 50, 71122 Foggia, Italy; 6 Department of General Surgery and Medical-Surgical Specialties, Dental Clinic, Unit of Orthodontics, University of Catania, Catania, Italy; 7 Department of Orthodontics and Pediatric Dentistry, University of Michigan School of Dentistry, Ann Harbor, MI USA

**Keywords:** Miniscrew, Skeletal anchorage, Maxillary expansion

## Abstract

**Introduction:**

Maxillary expansion in patients at the end of their growth relies on the possibility to use miniscrew supported expanders to apply expansion forces directly to the midpalatal suture. Although miniscrews provide a stable anchorage unit, several studies have reported that they do not remain in exactly the same position during treatment. The aim of the present study was to analyze miniscrew position changes after the expansion using bone-borne appliances in late adolescent patients.

**Methods:**

Nineteen patients (13 females, 6 males), with a mean age of 17.81 (SD = 4.66), were treated with a Bone-Borne Expander Device. The appliance was designed with 4 miniscrews: 2 in the anterior palatal area, at the third rugae level; 2 in the posterior area. A CBCT and an intraoral scan were obtained before treatment (T0), and then, a second CBCT was obtained after the expansion (T1). Data on peri-suture bone thickness were collected at T0, then the CBCTs were superimposed, and changes between mini-screws position on T0 and T1 were evaluated, both by linear and angular displacements.

**Results:**

Significant longitudinal differences were found in the distance of the head and the tip of miniscrews measured at the occlusal plane, as well as angular changes. Correlations between displacement measurements and peri-suture bone thickness and height measurements were found as well.

**Conclusions:**

While acting as bone anchor units, miniscrews do not remain in the same position during bone-borne expansion. The amount of displacement was related to peri-sutural total bone height and cortical thickness, especially in the anterior area of the naso-frontal maxillary complex.

## Introduction

In the last few years, an increased interest in maxillary expansion in patients at the end of their growth has been observed. Clinical studies have shown the effect of different appliances used to open the midpalatal suture in adults, young adults and late adolescent patients [1–3].

This indication relies on the possibility to use miniscrews supported expanders; these appliances can be named differently according to the configuration, to the support and generally are defined as bone-borne appliances, where the expansion screw dissipates its force through the miniscrews only. Hybrid appliances include in the support also teeth other than miniscrews [4].

Also, the clinical procedures can differ and include “appliance first approach” [5] or alternatively “miniscrew first approach”, introduced by Wilmes et al. [6]

Generally, the latter includes digital planning and the use of 3D-printed surgical guides [7].

Previous studies have analyzed the effects of the aforementioned appliances on the skeletal transversal changes, dental movements or airways improvement [2, 8]. Winsauer et al. findings indicated that, even in late adolescence, a skeletal expansion can be achieved with a success rate reported as high as 84.4% [9]. Interestingly, few analyses were conducted on miniscrews movement when orthopedic forces are used [10].

Miniscrews position under orthodontic load has been analyzed, during treatment with distal-screw appliance for example a change of inclination was observed due to continuous loading [11], even though they allowed a stable anchorage [12].

Up to now, miniscrew position changes after the expansion using bone-borne appliances in late adolescent patients have not been investigated and may lead to dissipation of the expansion forces or change in the direction of force application on the sutures. Thus, the aim of the present high-resolution CBCT report is to analyze three-dimensional changes of miniscrews position during maxillary expansion in patients treated with a bone-borne appliance. The hypothesis of the study was the null hypothesis, i.e., that there would be no differences over time in the parameters describing the movement of the miniscrews.

## Materials and methods

This retrospective study received Ethical Committee Approval (n° 2022/51 from Genova university, Italy) and included a sample of 19 consecutively treated patients (13 females, 6 males), with a mean age of 17.82 (SD = 4.66, minimum and maximum age 13 and 29 years, respectively). Patients included in the study respected the following inclusion criteria: no systemic disease, no previous orthodontic treatment, no alteration of bone metabolism, transverse maxillary deficiency with unilateral or bilateral posterior crossbite, permanent dentition including second molar eruption, no surgical or other treatment that might influence the rapid maxillary expansion outcome during the expansion procedure.

Transverse maxillary deficiency was evaluated using as reference the right and left most concave point on vestibule at the level of the mesio-buccal cusp of first molars in the maxilla and the distance between left and right WALA ridge in the mandible [13].

## Clinical procedures

All patients were treated with an orthodontic protocol in which the first step was the maxillary expansion thanks to a Bone-Borne Expander Device.

The appliance was designed with 4 miniscrews: 2 in the anterior palatal area (the shorter miniscrews), at the third ruga level; 2 in the posterior area (the longer miniscrews), between second premolar and first molar area, where the root distance is more favorable, approximately at a distance of 6–8 mm from the alveolar crest.

If anatomic conditions prevented such an ideal position, an alternative extraradicular site was selected at the level of the second premolar between the nasal and sinus cortical.

A CBCT was obtained before treatment (T0), and a second CBCT was obtained after the expansion (T1) to evaluate proper skeletal expansion and suture opening. Also, an intraoral scan was obtained before treatment.

Each miniscrew (9 mm and 15 mm length, diameter 2 mm, Spider Screw; HDC, Thiene, Italy) position was planned using Dolphin software module (three-dimensional module; Dolphin Imaging & Management Solutions, Chatsworth, Calif); the .stl file of the intraoral scan was imposed at the first CBCT, overlapping the model’s details to the dental-skeletal profile of the CBCT itself.

**Fig. 1:**
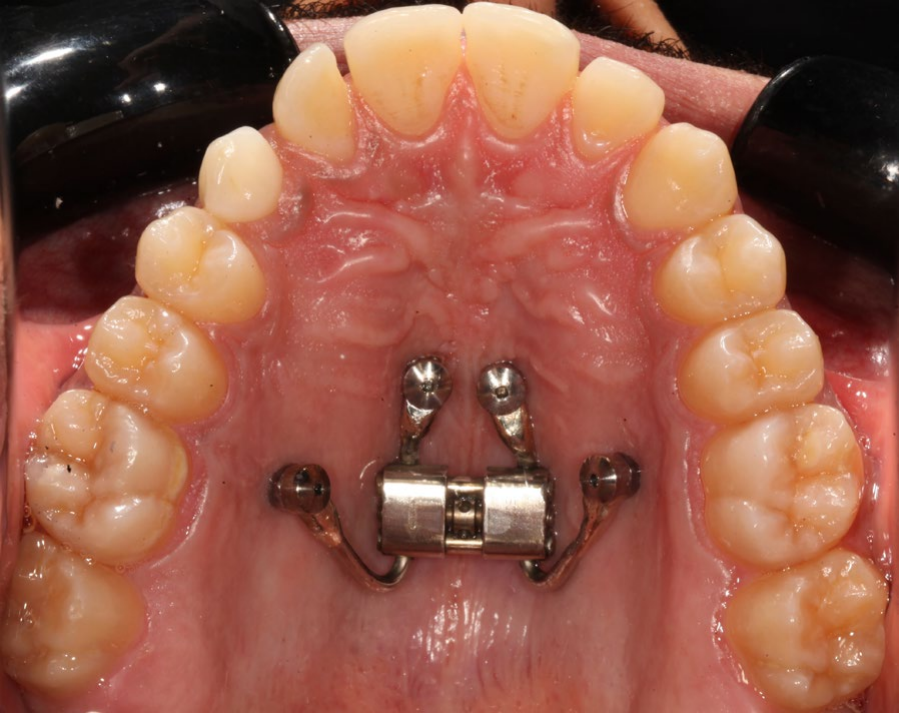
Appliance in situ before expansion phase

For each patient, 2 insertion guides were designed and three-dimensionally printed (Form 2; Formlabs, Sommerville, Mass), including 2 sleeves each in a cross position, each guide allowing the insertion of 2 miniscrews. After a chlorhexidine gluconate oral rinse, a preliminary guide fitting check was performed, and thereafter, local anesthesia was applied in correspondence with the palatal insertion sites. To improve procedure precision and surgery ergonomics, guides were fixed to the teeth using a fluid resin (trial gel; Dentsply GAC International, Islandia, NY); all the miniscrew insertions were preceded by pilot drill use for a cortical perforation. A dedicated pickup instrument was used to attain the correct depth stop indication planned with the digital insertion procedure. All screws were inserted with an insertion torque that was between 15 and 30 Ncm using a low-speed handpiece. After the guide removal, the palatal surface was cleaned with a physiological solution, and the bone-borne expander was inserted. The activation protocol was 2 turns per day until reaching the desired expansion. The device remained for another 12 months after the end of the expansion for all patients (Fig. 1).

### Miniscrew position analysis

For the mini-screw position analysis, the CBCTs performed before and after treatment and the intraoral scan with the screw planning were used.

First of all, CBCTs were superimposed.

The generated DICOM files were converted to NIFTI files thanks to the open-source software ITK-SNAP. Before starting the steps described below, every CBCT was downsized to standardize the image resolution and avoid any heterogeneity of the images and ease the superimposition steps. Thanks to “Downsize image” tools voxel size (spacing) was set at 0.5 mm^3^.

All the image analysis steps were performed by one operator as follows:

1. Construction of 3D volumetric label maps (segmentation) and 3D surface models of T0 scans: automatic segmentations were generated in the 3D Slicer software using the “Segment Editor” extension.
2. Head Orientation using “Transforms” extension: T0 CBCT (.nifti) and T0 Segmentation (.stl) were loaded on the 3D Slicer software. The software provides a fixed 3D coordinate system with three orthogonal planes denoted by yellow, red and green colors, representing sagittal, axial and coronal planes, respectively. These planes were used as a reference to orient (translate and or rotate) the T0 model of each patient using Glabella, Crista Galli and Basion to define the midsagittal plane, and bilateral structures of Orbitale and Porion (Frankfort horizontal plane) utilized to define the axial plane
3. Manual approximation: T0 and T1 CBCTs (.nifti) were loaded on the 3D Slicer software. Using the “Transform” extension, the T1.nifti scans were translated and rotated manually to superimpose them to T1 anterior cranial bases.
4. Construction of 3D volumetric label maps of approximated T1 scans: the same procedure described in step #1 was used to construct T1 segmentations.
5. Voxel-based registration of T0 and T1 scans using cranial base as reference: 3D voxel-based registration (“CMF Reg” extension in the 3D Slicer software) was used to align the T0 and T1 scans automatically by using corresponding voxels in the cranial base to achieve a reliable and reproducible superimposition of the two time point scans of each patient. Once this automated voxel-based registration was completed, the registered files (scans and segmentations) were used for subsequent steps.
6. The T1 registered scans were used by ITK-SNAP open-source software to create a virtual three-dimensional model of mini-screws only. This process, called segmentation, required outlining the shape of the mini-screws visible in the slices, setting up a threshold of the tissue density in order to select the only structures of interest.
7. The T0 planning model STL file was superimposed to T0 oriented scan on 3D Slicer software thanks to “Registration Wizard” tools. Some registration landmarks were placed on the teeth cusps of the STL model and on the same cusps of the scan segmentation. Thanks to an automated process, the planning model translates on the T0 scan.

**Fig. 2:**
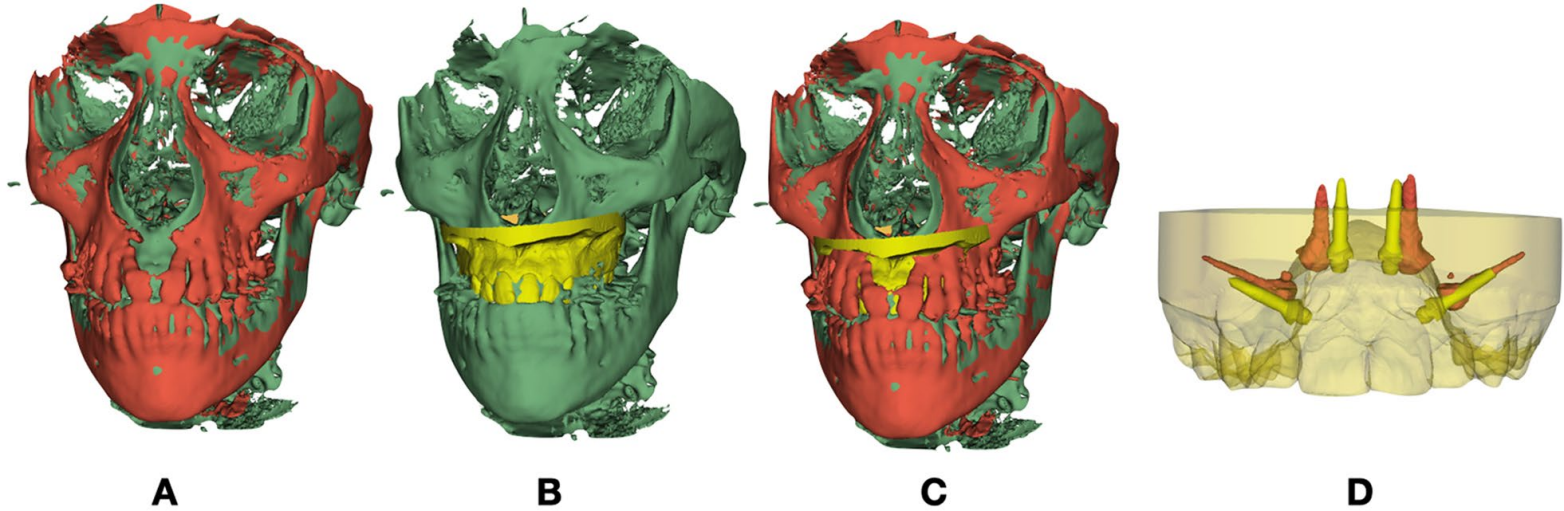
**A** Superimposition of T0 (green) and T1 (red) CBCT segmentation. **B** Superimposition of planning model (yellow) on previously oriented T0 CBCT Segmentation. **C** Superimposed CBCTs, T1 Screw Segmentation and planning model. **D** Screws at T0 and T1. Previously superimposed CBCT gave images of screws at T0 and T1

Thanks to the previous CBCTs superimposition, the planning model and the mini-screws segmentation are accordingly superimposed (Fig. 2).

Reference structures on the previously superimposed CBCTs and models are then used as stable reference parameters for the subsequent measurements.

Occlusal plane, taken on T0 superimposed planning models, and Midline plane, taken on the T0 superimposed CBCT, were used as reference structures.

Two planes were traced on 3D Slicer software: the Occlusal Plane (OP) related to T0 planning models; the Midline plane (MP) related to T0 CBCT.

Changes between mini-screws position on T0 and T1 were evaluated, both by linear movement and angular ones.

Linear and angular measurements were taken from an operator with 3D Slicer software thanks to the “Q3DC measurements” tool.

Landmarks were placed on all mini-screws heads and tips both on T0 time point (planning model) and on T1 time point (mini-screws segmentation).

**Fig. 3:**
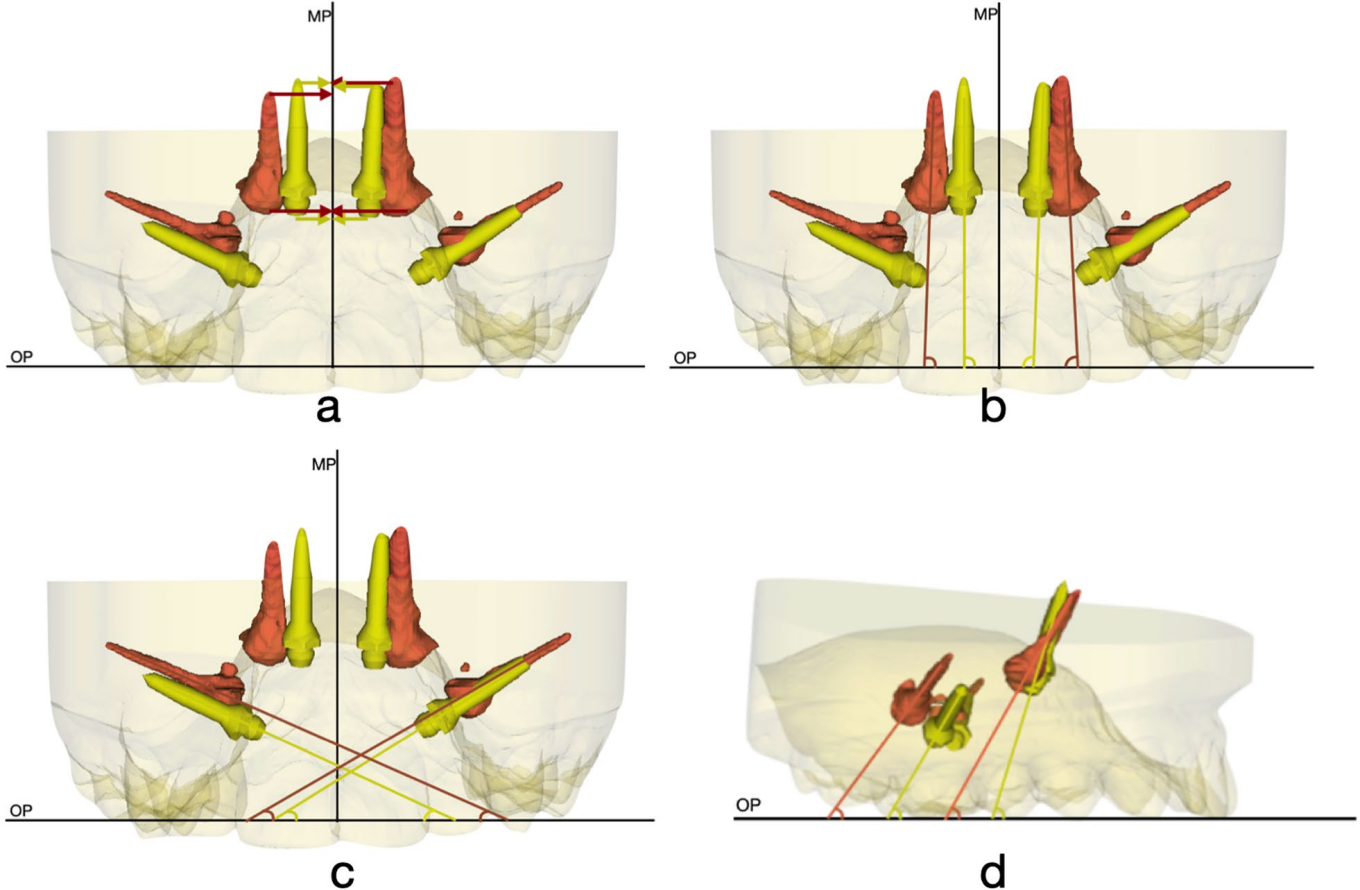
**a** Distance between T0 and T1 screw head and tip and MP. **b** Angular measurements between anterior screw and OP in frontal view. **c** Angular measurements between posterior screw and OP in frontal view. **d** Angular measurements between anterior and posterior screw and OP in lateral view

Linear measurements were taken from anterior mini-screws head and the MP and from anterior mini-screws tip and the MP (Fig. 3a).

Angular measurements were taken from every mini-screws axis both on T0 and T1 and OP. Measurements were evaluated from two different points of view in order to analyze two different angles. Measurements were taken on frontal view and also in lateral right view (Fig. 3).

Then asymmetrical expansion was evaluated by linear distances of 4 anatomical points to the MP plane in the T0 and T1 CBCT segmentations. The anatomical points were: Anterior Nasal Spine (ANS) at Right (ANS-R) and Left (ANS-L) and Posterior Nasal Spine (PNS) at Right (PNS-R) and Left (PNS-L).

**Fig. 4:**
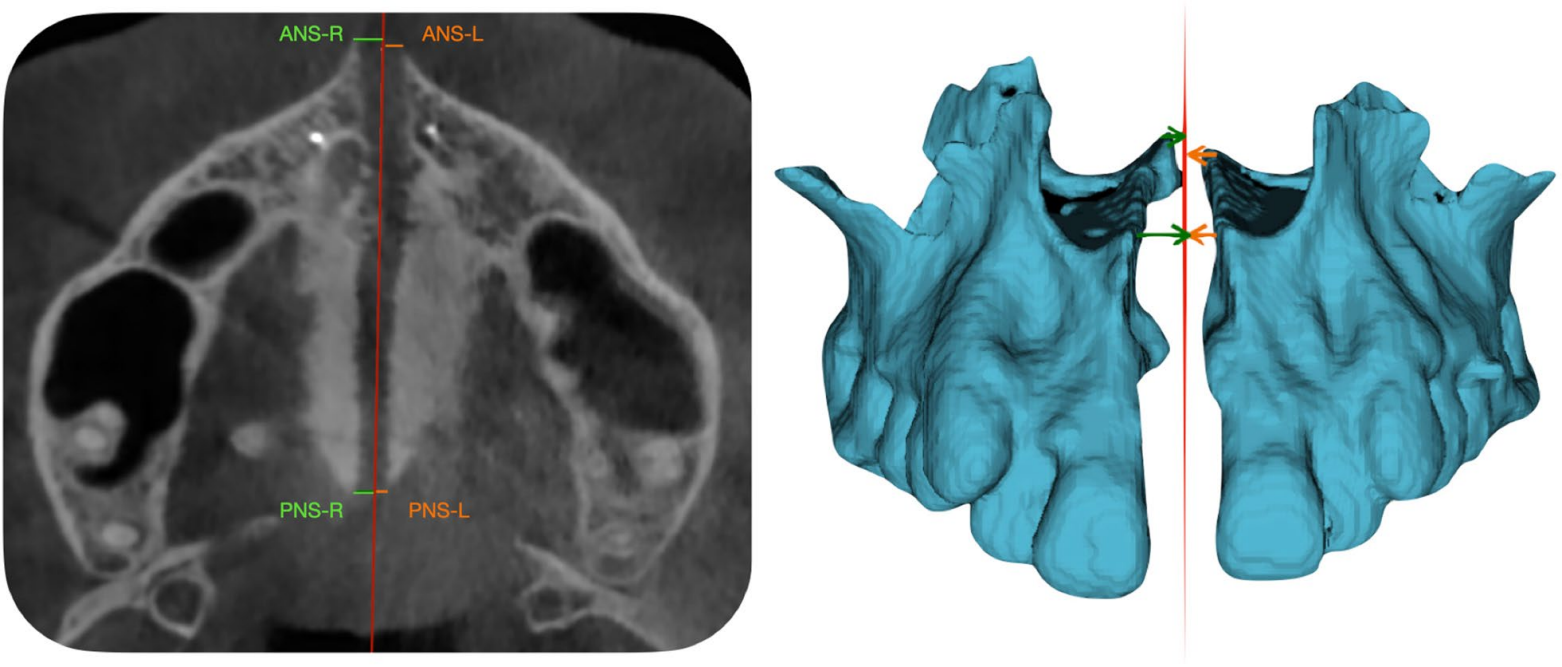
Measurements of skeletal asymmetry after maxillary expansion with MARPE. Measurements were taken on T1 CBCT (previously superimposed to T0 CBCT). Midline was taken on T0 CBCT

Landmarks were placed on these anatomical points (ANS-R, ANS-L, PNS-R, PNS-L) and distance between MP, and each point was performed (Fig. 4).

### Peri-suture bone measurements

**Fig. 5:**
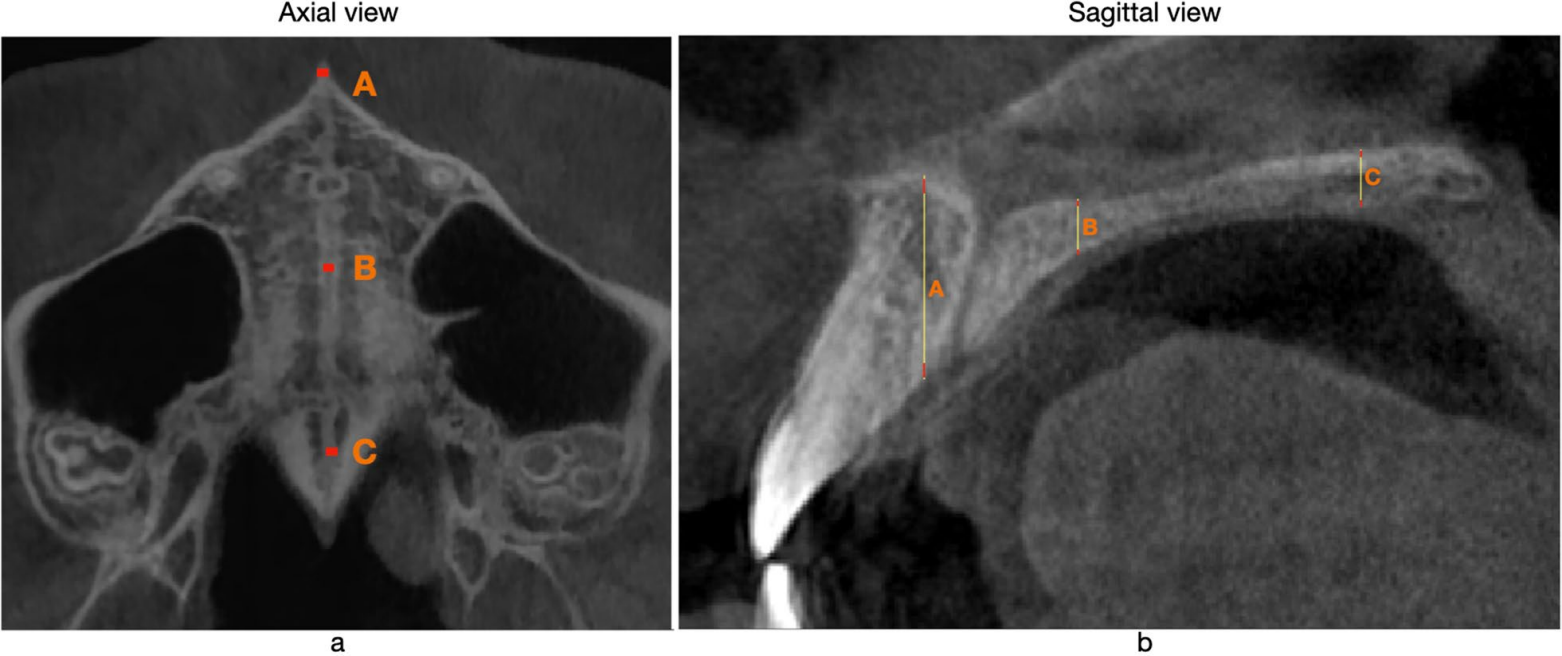
**a** In axial view, three areas were identified. A is the Anterior area; B is the Middle area and C is Posterior area. **b** In sagittal view, bone thickness was measured in the 3 zones described above. Distance between Right and Left cortical bone at suture was taken. Measurements in the coronal view are not shown

Possible correlations between miniscrews position changes with anatomical bone characteristics were evaluated measuring maxillary width and bone thickness for each patient in the CBCT performed at T0 time at different levels. All measurements were carried out at three different levels: Anterior zone (at midway from nasopalatine canal and Anterior Nasal Spine), Middle zone (between second upper premolar and first upper molar), Posterior zone (10 mm anterior to Posterior Nasal Spine). These points were identified in an axial view. In this view were carried out linear measurements of midpalatal suture thickness (Fig. 5a). In the Sagittal View, entire bone thickness measurements were carried out as well as the two cortical thickness (palatal and nasal). These measurements were taken in the 3 zones previously identified (Anterior, Middle and Posterior zone) (Fig. 5b). The inclination of the alveolar process was evaluated in a coronal view, by observing the angle between the tangent to the palatal side of the alveolar bone and the nasal floor plane (medial angle).

## Sample size

The sample size estimation calculated that 19 patients would achieve 80% power to detect a mean difference over time in the miniscrew angle to occlusal plane of 5°, with an assumed standard deviation of differences of 5.5°, and with a significance level (alpha) of 0.05 using a *t* test. The sample size calculation was performed on the basis of results from a previous pilot study (unpublished data).

### Statistical analysis

To verify the normality of the data, the Shapiro–Wilk test was used. Continuous variables are given as means ± standard deviations (SD) and medians with interquartile range (IR), whereas categorical variables as number and/or percentage of subjects.

Differences in the linear and angular measurements between timepoints were tested by the Student’s paired *t* test or Wilcoxon’s signed rank test adjusted by using Bonferroni method. The comparison between right and left side for the longitudinal differences of the miniscrew position measurements was tested again by the Student’s paired *t* test or Wilcoxon’s signed rank test adjusted by using Bonferroni method.

The correlation between the miniscrew axis angle longitudinal difference measured in the frontal view and the peri-suture cortical thickness measurements in the coronal view at the anterior, 5–6 or posterior zone, the homolateral measured alveolar bending and the cortical thickness measurements in the sagittal view was tested by the Pearson’s correlation test, or Spearman’s rank correlation test.

Differences with a *p* value < 0.05 were selected as significant. Data were acquired and analyzed in R v3.4.4 software environment [14].

## Results

The null hypothesis was rejected: significant longitudinal differences (i.e., differences over time) were observed in the inter-screw distance at the head level and in the distance of the head and the tip of each anterior miniscrew from the Perpendicular to occlusal plane passing through midline (POP) (*p* < 0.001, Table 1). The same holds for posterior miniscrews at the head level (*p* < 0.001), and for the tip of the posterior right miniscrew (*p* = 0.020, Table 1).

**Table 1:** Descriptive statistics of the different linear and angular miniscrew measurements at baseline (T0) and T1

	T0	T1	*p* value
Frontal view			
Anterior miniscrews—interhead distance (mm)	6.09 ± 0.61	13.39 ± 2.07	< 0.001*
Right anterior miniscrew—head to POP distance (mm)	2.82 ± 1.25	6.14 ± 1.35	< 0.001*
Left anterior miniscrew—head to POP distance (mm)	3.24 ± 1.32	7.51 ± 2.05	< 0.001*
Anterior miniscrews- intertip distance (mm)	6.19 ± 0.64	10.78 ± 2.18	< 0.001*
Right anterior miniscrew—tip to POP distance (mm)	3.07 ± 0.89	5.69 ± 1.05	< 0.001*
Left anterior miniscrew—tip to POP distance (mm)	3.18 ± 1.13	5.35 ± 1.97	< 0.001*
Posterior miniscrews- interhead distance (mm)	15.89 ± 3.16	24.04 ± 3.56	< 0.001*
Right posterior miniscrew—head to POP distance (mm)	7.58 ± 1.72	11.87 ± 1.95	< 0.001*
Left posterior miniscrew—head to POP distance (mm)	8.23 ± 2.56	12.27 ± 2.90	< 0.001*
Posterior miniscrews- intertip distance (mm)	44.72 ± 4.93	46.37 [43.37, 48.61]	0.418
Right posterior miniscrew—tip to POP distance (mm)	23.09 [19.73, 24.38]	23.32 ± 2.39	0.020*
Left posterior miniscrew—tip to POP distance (mm)	22.97 ± 2.69	23.15 ± 2.75	0.734
Right posterior miniscrew—angle to occlusal plane (°)	35.63 [34.56, 37.33]	37.91 [36.34, 43.99]	0.055
Left posterior miniscrew—angle to occlusal plane (°)	33.61 ± 5.76	43.26 [33.79, 45.27]	0.002*
Right anterior miniscrew—angle to occlusal plane (°)	87.99 ± 1.58	87.34 [85.24, 87.75]	0.011*
Left anterior miniscrew—angle to occlusal plane (°)	88.09 ± 1.44	83.30 [80.02, 85.62]	< 0.001*
Lateral view			
Right anterior miniscrew—angle to occlusal plane (°)	67.19 ± 8.23	66.90 ± 9.58	0.780
Left anterior miniscrew—angle to occlusal plane (°)	66.95 ± 7.97	67.32 ± 11.47	0.771
Right posterior miniscrew—angle to occlusal plane (°)	63.81 ± 7.73	65.20 [61.25, 71.34]	0.417
Left posterior miniscrew—angle to occlusal plane (°)	66.54 ± 9.71	68.70 [51.13, 77.30]	0.899

POP, perpendicular to occlusal plane. Results are expressed as Mean ± Standard Deviation or Median [Interquartile Range]. *P* value: paired* t* test, or Wilcoxon signed rank test, *p* value. *statistically significant

A significant longitudinal difference of 4.94 (IR = [2.93, 9.15]) degrees was found for the angle formed by the posterior left miniscrew with respect to the occlusal plane (*p* = 0.002, Table 1).

A significant longitudinal difference of − 1.72 (IR = [− 2.31, − 0.27]) degrees was found for the angle formed by the anterior right miniscrew with the occlusal plane (*p* = 0.011, Table 1).

A significant longitudinal difference of − 4.90 (IR = [− 8.42, − 2.03]) degrees was found for the angle formed by the anterior left miniscrew with the occlusal plane (*p* < 0.001, Table 1), and this variation was significantly different from the one encountered by the right miniscrew (*p* = 0.026, Table 2).

**Table 2:** Descriptive statistics of the different linear and angular longitudinal differences

	Right side	Left side	*p* value
Frontal view			
Anterior miniscrews—head to POP distance (mm)	3.31 ± 1.48	4.26 ± 1.95	0.071
Anterior miniscrews—tip to POP distance (mm)	2.61 ± 1.16	2.16 ± 1.56	0.259
Posterior miniscrews—head to POP distance (mm)	4.29 ± 1.58	4.03 ± 1.70	0.578
Posterior miniscrews—tip to POP distance (mm)	1.32 ± 2.14	− 0.48 [− 1.81, 2.01]	0.182
Posterior miniscrews—angle to occlusal plane (°)	3.21 ± 6.78	5.05 ± 5.68	0.264
Anterior miniscrews—angle to occlusal plane (°)	− 1.86 ± 3.08	− 4.90 [− 8.42, − 2.03]	0.026*
Lateral view			
Anterior miniscrews—angle to occlusal plane (°)	− 0.28 ± 4.54	0.40 ± 5.60	0.522
Posterior miniscrews—angle to occlusal plane (°)	0.94 [− 0.93, 5.82]	0.48 [− 3.50, 8.42]	1.000

POP, perpendicular to occlusal plane. Results are expressed as Mean ± Standard Deviation or Median [Interquartile Range]. *P* value: paired* t* test, or Wilcoxon signed rank test, *p* value. *statistically significant

The peri-suture bone measurements are shown in Table 3.

**Table 3:** Descriptive statistics of the different peri-suture bone measurements at baseline. Results are expressed as Mean ± Standard Deviation or Median [Interquartile Range]

Frontal view	
Suture thickness of the anterior area measured in the coronal view (mm)	1.37 ± 0.59
Suture thickness of the middle area measured in the coronal view (mm)	1.31 ± 0.83
Suture thickness of the posterior area measured in the coronal view (mm)	1.25 ± 0.46
Alveolar inclination to nasal floor (°)	1.57 ± 3.51
Palatal cortical thickness in the anterior area measured in the sagittal view (mm)	1.19 ± 0.50
Nasal cortical thickness in the anterior area measured in the sagittal view (mm)	0.91 ± 0.24
Total bone height in the anterior area measured in the sagittal view (mm)	15.53 ± 2.95
Palatal cortical thickness in the median area measured in the sagittal view (mm)	0.80 ± 0.20
Nasal cortical thickness in the median area measured in the sagittal view (mm)	0.76 ± 0.22
Nasal cortical thickness in the posterior area measured in the sagittal view (mm)	0.78 ± 0.27
Palatal cortical thickness in the posterior area measured in the sagittal view (mm)	0.77 ± 0.27
Total bone height in the posterior area measured in the sagittal view (mm)	4.36 ± 1.62

A positive correlation was found between the angle variation occurring to the posterior left miniscrew and the distance of the PNS-L point to the midline (*r* = 0.682, *p* = 0.003, Table 4).

**Table 4:** Correlation between the miniscrew axis angle longitudinal difference measured in the frontal view and the distance from the median palatine process to the midline measured at AND or PNS level

	AND_Right		AND_Left		PNS_Right		PNS_Left	
	*r*	*P* value	*r*	*P *value	*r*	*P *value	*r*	*P *value
Frontal view								
Left anterior miniscrew—angle to occlusal plane	0.154	0.553	0.108	0.680	0.244	0.345	0.336	0.203
Right anterior miniscrew—angle to occlusal plane	− 0.099	0.706	− 0.44	0.866	− 0.203	0.435	− 0.017	0.949
Left posterior miniscrew—angle to occlusal plane	0.293	0.254	0.36	0.156	0.267	0.301	0.682	0.003*
Right posterior miniscrew—angle to occlusal plane	0.048	0.053	0.228	0.379	0.463	0.061	0.404	0.121

*r*, Pearson correlation coefficient, or Spearman’s rank correlation test coefficient; *P *value, *P *value of Pearson correlation test, or *P *value of Spearman’s rank correlation test coefficient. *statistically significant

A negative correlation was found between the angle variation occurring to the posterior right miniscrew and the cortical thickness at the suture level measured in the frontal view at the posterior area (*r* = − 0.607 *p* = 0.013, Table 5).

**Table 5:** Correlation between the miniscrew axis angle longitudinal difference measured in the frontal view and the peri-suture cortical thickness measured in the frontal view at the anterior, middle or posterior zone and the homolateral measured alveolar bending

	CRVTHANT		CRVTHMED		CRVTHPOST		ProcAlv/NF	
	*r*	*P *value	*r*	*P *value	*r*	*P *value	*r*	*P *value
Frontal view								
Left anterior miniscrew—angle to occlusal plane	0.046	0.867	− 0.072	0.791	0.068	0.805	0.203	0.528
Right anterior miniscrew—angle to occlusal plane	0.242	0.367	0.387	0.139	− 0.021	0.943	− 0.371	0.235
Left posterior miniscrew—angle to occlusal plane	− 0.251	0.348	− 0.0839	0.758	− 0.209	0.438	− 0.017	0.959
Right posterior miniscrew—angle to occlusal plane	− 0.16	0.555	− 0.34	0.198	− 0.607	0.013*	0.229	0.473
Right anterior miniscrew—head to occlusal plane distance	0.067	0.805	− 0.103	0.703	− 0.147	0.586	0.388	0.212
Left anterior miniscrew—head to occlusal plane distance	0.344	0.192	0.48	0.060	0.28	0.294	− 0.010	0.974

	ANTPTH		ANTNTH		ANTTOTHEI		MEDPTH	
	*r*	*P *value	*r*	*P *value	*r*	*P *value	*r*	*P *value
Frontal view								
Left anterior miniscrew—angle to occlusal plane	0.245	0.444	− 0.368	0.24	− 0.217	0.499	− 0.032	0.905
Right anterior miniscrew—angle to occlusal plane	− 0.343	0.275	− 0.287	0.366	− 0.783	0.004*	0.1	0.712
Left posterior miniscrew—angle to occlusal plane	− 0.736	0.006*	− 0.708	0.01*	− 0.343	0.275	0.343	0.194
Right posterior miniscrew—angle to occlusal plane	0.029	0.928	− 0.311	0.326	0.336	0.286	− 0.047	0.862
Right anterior miniscrew—head to occlusal plane distance	0.010	0.976	0.096	0.767	0.365	0.243	0.021	0.938
Left anterior miniscrew—head to occlusal plane distance	− 0.245	0.443	0.087	0.788	− 0.128	0.691	0.207	0.442

	MEDNTH		POSTNTH		POSTPTH		POSTTOTHEI	
	*r*	*P *value	*r*	*P *value	*r*	*P *value	*r*	*P *value
Frontal view								
Left anterior miniscrew—angle to occlusal plane	− 0.073	0.788	0.362	0.169	0.124	0.648	− 0.059	0.831
Right anterior miniscrew—angle to occlusal plane	0.053	0.848	0.279	0.294	0.258	0.336	− 0.006	0.987
Left posterior miniscrew—angle to occlusal plane	0.267	0.317	0.517	0.040*	0.409	0.116	− 0.097	0.719
Right posterior miniscrew—angle to occlusal plane	0.047	0.863	0.028	0.917	− 0.026	0.922	0.044	0.872
Right anterior miniscrew—head to occlusal plane distance	− 0.131	0.629	0.355	0.177	0.298	0.262	− 0.146	0.59
Left anterior miniscrew—head to occlusal plane distance	0.152	0.574	− 0.068	0.802	0.010	0.713	− 0.117	0.667

*r*: Pearson correlation coefficient, or Spearman’s rank correlation test coefficient; *P *value: *P *value of Pearson correlation test, or *P *value of Spearman’s rank correlation test coefficient. *statistically significant

CRVTHANT, suture thickness of the anterior area measured in the coronal view; CRVTHMED, suture thickness of the middle area measured in the coronal view; CRVTHPOST, suture thickness of the posterior area measured in the coronal view; ProcAlv/NF, alveolar inclination to nasal floor; ANTPTH, palatal cortical thickness in the anterior area measured in the sagittal view; ANTNTH, nasal cortical thickness in the anterior area measured in the sagittal view; ANTTOTHEI, total bone height in the anterior area measured in the sagittal view; MEDPTH, palatal cortical thickness in the median area measured in the sagittal view; MEDNTH, nasal cortical thickness in the median area measured in the sagittal view; POSTNTH, nasal cortical thickness in the posterior area measured in the sagittal view; POSTPTH, palatal cortical thickness in the posterior area measured in the sagittal view; POSTTOTHEI, total bone height in the posterior area measured in the sagittal view

A negative correlation was found between the angle variation occurring to the anterior right miniscrew and the total bone height measured in the sagittal view at the anterior area (*r* = − 0.785 *p* = 0.004, Table 5).

A positive correlation was found between the angle variation occurring to the posterior left miniscrew and the nasal bone thickness measured in the sagittal view at the posterior area (*r* = 0.517 *p* = 0.040, Table 5).

A synopsis of different sutural and device transversal measurements is reported (Table 6).

**Table 6:** Synopsis of different sutural and device transversal measurements

Screw nominal expansion (mm)	Intercrestal diameter variation (furcation of the sixth) (mm)		Miniscrews-interhead distance variation (mm)	Suture opening (sum of the left and right distance to the midline) (mm)	Bisp (mm)	Asymmetry (absolute value of the difference between the left and right distance to the midline) (mm)	Suture opening/screw nominal expansion	Bisp/screw nominal expansion
9.11 ± 1.60	5.07 ± 1.70	Anterior	7.30 ± 2.29	5.60 ± 2.24	4.77 ± 1.71	1.03 ± 0.56	61.5%	52.4%
		Posterior	8.15 ± 2.76	2.72 ± 1.60	2.97 ± 1.55	0.62 ± 0.51	29.9%	31.9%

**Fig. 6:**
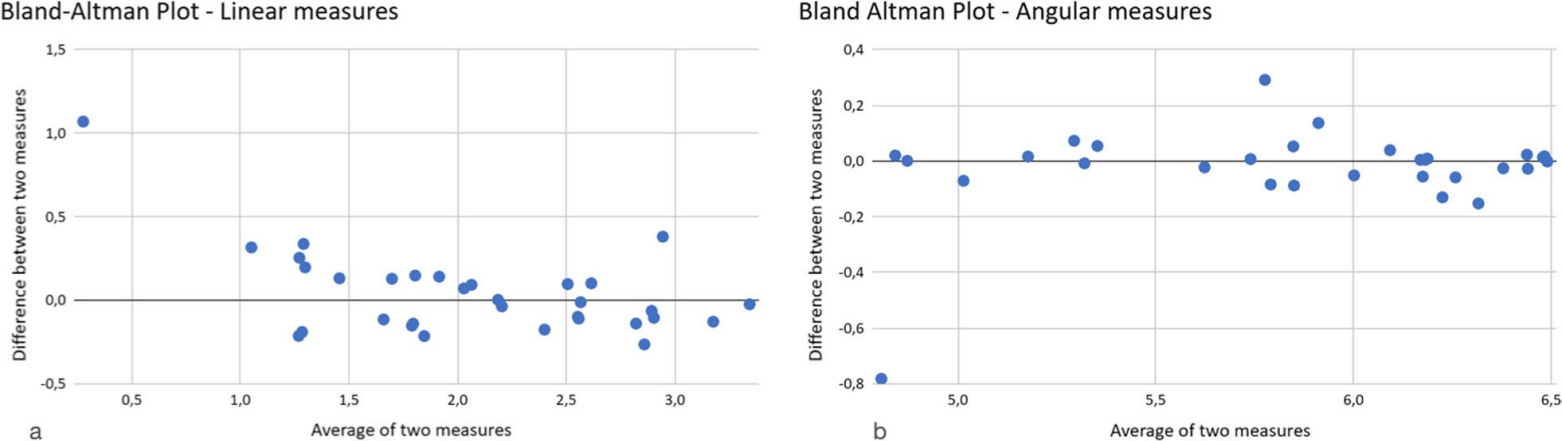
**a** Bland–Altman plot—linear measures. **b** Bland–Altman plot—angular measures

An intra-observer error assessment by using a Bland–Altman analysis over a sample of 32 linear measurements and 32 angular measurements in the frontal view was performed. The mean difference between the measures was 0.04 and − 0.02 for linear and angular measurements, respectively. The 95% limits of agreement were [− 0.46, 0.54] for linear measurements and [− 0.34, 0.29] for angular measurements (Fig. 6a, b).

## Discussion

Orthopedic maxillary expansion in late adolescent or adult patients when a transverse defect has been diagnosed has been delegated for years to maxillofacial surgeons. The surgical-assisted rapid palatal expansion (SARPE) is a valuable therapeutic procedure that remains always reliable even though some side effects have been reported, mainly appliance related [15, 16].

The literature has already described that, even though miniscrews act as a stable anchorage for orthodontic tooth movement, they do not remain absolutely stationary like an endosseous implant throughout orthodontic loading and might move according to the orthodontic loading in some patients [17, 18].

According to the present study, all miniscrews used for bone-borne expansion underwent angular displacement up to 11° for posterior screws and 8° for anterior screws. One anterior screw underwent 39° variation because of loosening of connection screws during activation. Moreover, asymmetry of miniscrew position changes was observed: both left screws showed a greater amount of displacement and angular variation when compared to the opposite side; this observation suggests a greater stability loosening on the left side in respect to the right side. These results can be related to the asymmetrical skeletal expansion results observed in a previous study, where the right side showed more displacement when compared to the left side [19].

The observed displacement may rely on several factors, such as the miniscrew diameter, orthodontic load magnitude, depth of the miniscrew inside the bone, bone quality and quantity at the implant site. The present study took into consideration the bone characteristic of the naso-maxillary complex as well, by providing peri-suture bone thickness measurements and total bone height in anterior and posterior area. Particularly, it was found that total bone height measured in the sagittal view at the anterior area was negatively related to the angle variation occurring to the anterior right miniscrew. The interpretation of the result is complicated by the fact that angular variation in the anterior area is expressed by a negative number because of the reading verse of angles provided by the used software, but the meaning of the correlation is that the greater the total bone height, the greater the angle variation, and this type of association holds for palatal and nasal cortical parameters as well. In other words, we may look at these osseous measurements as an indirect measure of the resistance of the system and of the stress developed at the miniscrews level. These findings are in accordance with a previous study of De Jesus et al., stating that a lower palatal bone thickness in the area from 12 to 16 mm posterior to the incisive foramen would represent a relevant factor in the opening of the suture and is presumably related to lower resistances [20].

Data observed from the present research suggest from a clinical point of view that the greater the cortical thickness and total bone height, the lower will be the screw displacement; in other words, it seems that the screw’s position remains more stable in these conditions and could more effectively act as maximum anchorage during expansion.

Another factor related to miniscrew displacement is the waiting period. In a study on forty-one miniscrews, buccal, palatal and midpalatal mini-implants showed some displacement (mean value ≤ 0.78 mm) when submitted to force after a 5 months period, although a comparison with the present study is not completely reliable because the used superimposition CBCT method was different [21]. Moreover, the present study describes miniscrew displacement under a midpalatal suture distraction procedure, which is characterized by an intense load in a relatively small timespan.

A systematic review of mini-implant displacement under orthodontic loading distinguishes between primary and secondary displacement. Primary displacement is intended as the immediate displacement of a miniscrew immediately loaded with force due to the elastic and plastic properties of the bone. Secondary displacement is defined as long-term displacement of a mini-implant loaded with force due to the remodeling processes of the bone [22].

Then, when considering the miniscrew-assisted orthopedic maxillary expansion period, we can infer from the literature that miniscrews involved in the expansion process undergo a loosening of the bone-to-screw contact due to bone viscoelastic properties and the ongoing bone remodeling cycle that could be eventually measured by a torque loss [23]. The effects of it lead both to primary and secondary displacement and could contribute to the dispersion of the activation energy of the expanding screw. In the present study, we did not take into account the screw linear displacement measures, but we can infer them from the measured angles and from linear measurements in the frontal view. The sum of these displacements should not exceed 1 mm; to calculate it, the length of the miniscrew and the after and before head to occlusal plane distance were considered, then the cotangent function on the angle variation was used. This assessment appears in accordance with the fact that the anterior miniscrew inter-head distance in the frontal view varies on average 7.3 mm between T1 and T0, and posterior miniscrew inter-head distance in the frontal view varies on average 8.15 mm, while the screw mean nominal expansion was 9.11 mm. Anyway, this does not explain completely why in front of a 9.11 mm opening of the screw we find only a 5.60 mm suture opening in the anterior area.

First, as a previous study by Moon et al. effectively described, the maxillary expansion pattern can be divided into naso-maxillary complex rotation, alveolar bone bending, and tooth tipping [2]. Particularly, the naso-maxillary complex was reported to open transversely in a pyramidal-like configuration on the coronal plane and the center of rotation would be the frontonasal suture. This pyramidal pattern suggests a reason why the expansion screw opening, which occurs at a lower height with respect to the pyramid vertex, is greater than the suture opening measure. However, in a more recent study it was observed that bone-borne expansion of the maxilla did not follow the pyramidal pattern reported in previous studies, with similar amounts of skeletal expansion observed at orbitale, zygoma and nasal cavity [19].

As a second source of “dispersion”, it was shown in the same study that one-half of the maxilla may move and displace more than the contralateral one, leading to an asymmetric palatal expansion; and the differences in the resistance of other circum-maxillary sutures may contribute to this effect, which in our study averaged 1 mm. Moreover, despite the almost null molar buccal tipping that has been reported for bone-borne appliances, the third factor that could play a role in absorbing the screw activation energy is alveolar bending that can absorb part of the screw positional changes [7]. Our findings also indicate a certain degree of bending of the expansion screw itself, due to the high resistance of the complex, and this would represent another cause of dispersion for the activation energy.

Finally, according to the present study the amount of expansion screw opening converted to sutural opening is estimated between 52.4% and 61.5% of the value in the anterior area and 29.9% and 31.9% of the value in the posterior area, being the anteroposterior difference due to the already known triangular (V-shaped) opening of the suture that was wider anteriorly [19].

There are some limitations that are useful to be underlined in the present report for a better understanding: more females than males were included in the sample, and this variable could have an influence on expansion quality and quantity. Every patient received a customized miniscrew insertion planning, this allowed a better identification of bone availability, but also represented not a perfect repeatable standard position even though the insertion areas were always the same. Moreover, the present study was based on the position of the screws in the planning model, and the mean self-parallelism loss of a screw between the planned position and the achieved one has been estimated between 3.74° and 4.68° by a recent study [24]; particularly, part of this parallelism would be already lost in the 3D-printed model. The linear displacement of the miniscrews did not exceed a unilinear mean value of 1.16 mm; a 0.44 mm mean difference in anterior miniscrew’s length measurements was found; this slight difference can be addressed to the segmentation phase and had a limited influence on the overall analysis.

## Conclusion

While acting as stable anchor units, miniscrews do not remain in the same position during bone-borne expansion. The amount of displacement appeared as related to peri-sutural total bone height and cortical thickness, especially in the anterior area of the naso-frontal maxillary complex. In the present study on miniscrew-assisted palatal expansion with a bone-borne device, the achieved expansion of the palatal vault was between 52.4% and 61.5% of the expansion screw opening.

## Data Availability

The datasets used and/or analyzed during the current study are available from the corresponding author on reasonable request.
